# The combined effects of temperature and salinity on the digestion and respiration metabolism of *Pinctada fucata*

**DOI:** 10.1038/s41598-022-26168-0

**Published:** 2022-12-14

**Authors:** Jingru Yang, Zhengyi Fu, Zhenhua Ma, Gang Yu

**Affiliations:** 1 Tropical Aquaculture Research and Development Center, South China Sea Fisheries Research Institute, Chinese Academy of Fishery Sciences, Sanya, Hainan People’s Republic of China; 2 Key Laboratory of South China Sea Fishery Resources Exploitation and Utilization, Ministry of Agriculture and Rural Affairs, Guangzhou, Guangdong People’s Republic of China; 3 Sanya Tropical Fisheries Research Institute, Sanya, Hainan People’s Republic of China; 4 College of Science and Engineering, Flinders University, Adelaide, South Australia Australia

**Keywords:** Marine biology, Animal physiology

## Abstract

The combined effects of temperature and salinity on the digestion and respiration metabolism of *Pinctada fucata* were evaluated via response surface methodology and box-benhnken design under laboratory condition. Results indicated that the primary and secondary effects of salinity and temperature had significant effects on amylase (AMS) of *P. fucata* (*P* < 0.05)., The digestive enzyme reached the maximum activity when temperature was 26 °C. The AMS and trypsin (TRYP) increased at first, and then decreased with increasing temperature. The Lipase (LPS) was positively correlated with either salinity or temperature. Salinity had no significant effect on TRYP as a primary effect (*P *> 0.05), but had a significant effect on TRYP as a secondary effect (*P *< 0.01). These effects were completely opposite to the effect of temperature on pepsin (PEP) as primary and secondary effects. The combined effects of salinity and temperature on AMS, TRYP and PEP were significant (*P *< 0.01), but had no significant effect on LPS (*P* > 0.05). The primary, secondary and interaction effects of salinity had significant effects on NKA (Na^+^-K^+^-ATPase) of *P. fucata* (*P* < 0.05), and NKA presented a U-shaped distribution with increasing salinity. The quadratic and interactive effects of temperature had a significant effect on AKP (*P* < 0.05), and AKP showed a U-shaped distribution with increasing temperature. Lactate dehydrogenase (LDH) activity decreased at first, and then increased when temperature and salinity changed from 20 to 30 °C and 23–33 ‰, respectively. The expression of GPX gene affected by temperature in gills may be delayed compared with that in hepatopancreas, and its expression is tissue-specific. The appropriate digestion and respiratory metabolism index models were established under the combined temperature and salinity conditions. The optimization results showed that the optimal combination of temperature and salinity was 26.288 °C/28.272‰. The desirability was 0.832. Results from the present study will provide a theoretical reference for shellfish culture affected by environmental interactions and the establishment of related index models.

## Introduction

Respiratory metabolism and digestion are no doubt run through the whole process of ontogenesis, during which organisms are exposed to changes in surrounding environmental factors. As a matter of common knowledge, aquatic organisms undergo synchronous impacts of a mass of environmental variables in their natural habitats^1^. Among them, salinity and temperature are the most important environmental factors affecting aquatic organisms, which can directly or indirectly regulate the rate of all bioprocesses, such as development, growth, survival and reproduction^1–3^. Salinity and temperature have therefore been described as dominant "ecological principal factor" for many marine species^1,4–7^.

Many auto-biologic studies on bivalves have mainly concentrated on single effects rather than multiple environmental factors, such as some studies conducted by Japanese researchers in the context of the influences of salinity and temperature on *P. fucata*^8–14^. Montgomery^15^ suggest that the interaction between interested factors are very important, and single factor experiments cannot provide relevant information about the interaction. As the report points out, the mass mortalities of Japanese pearl oysters since 1960s may have been posed by interactions of many factors and it has long been recognized that the influence of one factor can be modified by another factor^16^. During this period, many researchers used statistical methods (response surfaces) to conduct research.

Statistical methods were established by Box^17^ and first applied by Davis^18^ to look at ovum development, growth and larval survival of the Eastern oyster *Crassostrea virginia* and hard clam *Mercenaria mercenaria*. This methodology made it possible to study the synergy between multiple factors, under broader environmental conditions rather than under laboratory experiments. . Subsequently, the combined influences of the two factors have been investigated for numerous mollusks, such as mussel *Mytilus edulis*^19^, *Adula californiensis* (Pelecypoda: Mytilidae)^20^, clam *Rangia cuneata*^21^, *Mulinia lateralis*^22^, Northern Bay scallop *Argopecten irradians irradians*^23^, European flat oyster *Ostrea edulis*^24^, Mediterranean mussel *Mytilus galloprovincialis*, Pacific oyster *Magallana gigas*^25^, pearl oysters *Pinctada imbricata Röding*^16^, *Pinctada martensii*^1^, and noble scallop *Chlamys nobilis*^26^.

*Pinctada fucata*, is one of the chief shellfish varieties for cultivating seawater pearls worldwide^27^. Since 1949, *P. fucata* has been cultivated in Guangdong, Guangxi and Hainan Provinces of China, and the pearl yield reached its peak in the 1990s. This has profoundly promoted the development of pearl industry in China and created a respectable economical earning for the nation^28^. For example, the yield of seawater pearl in China reached six tons in 2010, for a gross value of about 20 million USD. Nevertheless, in the past 10 years, massive mortalities occurs in *P. fucata* have been frequently reported, resulting in enormous economic losses. A series of research have been conducted, followed by evidence that environmental changes, such as salinity, pH, dissolved oxygen, temperature and so on, are often associated with such mortalities^28–31^. Physiologically, aquatic organisms are affected by changes in ammonia excretion rate, osmotic pressure, cellular immune level and oxygen consumption rate and so on, resulting in disturbance of basal metabolic balance, inhibition of growth and death in severe cases^28,29,32–34^. Before large-scale farming of *P. fucata*, it is critical to identify the optimal conditions for such farms. To date, most studies consider one environmental factor at a time, while the existing multi-factor studies have focused on the growth, survival^35^, transcriptome, biomineralization^36^, energy budget^37^, immunological expression^38^, fertilization and hatching^1^. And Wang, et al.^1^, Wang, et al.^39^ and Wang, et al.^40^ studied the synergistic effects of salinity and temperature on *P. fucata*. As mentioned above, respiration metabolism and digestion throughout the whole process of ontogenesis are affected by changes in environmental factors.It is therefore necessary to explore in detail how salinity and temperature, in particular, jointly influence respiratory metabolism and digestion, and to determine in detail the optimal combination of factors with practical significance. Undoubtedly, insight into such issues will be beneficial for maintaining the optimal living conditions of *P. fucata*, assuring their growth, development and survival, thereby reducing economic losses and increasing production. The objectives of this study were to (1) examine the synergistic effects of salinity and temperature on respiratory metabolism and digestion in *P. fucata* using response surface method and Box-Benhnken design, (2) model the relationship of respiratory metabolism and digestion with salinity and temperature, (3) determine the optimal salinity–temperature combination using the statistical optimization technique. Results from the present study will provide a theoretical reference for shellfish culture affected by environmental interactions and the establishment of related index models.

## Results

### Model significance analysis

The regression equations of salinity and temperature on the digestive physiological indexes of *P. fucata* (each coefficient was the actual value) established in the experiment were as follows:

AMS = − 19.3181 + 0.6795 * T + 0.8290 * S − (9.8000E-003) * T * S − (7.8316E-003) * T^2 − 0.0102 * S^2

LPS = + 2.0610 − (6.7933E-003) * T + (5.0782E-003) * S − (2.6280E-005) * T * S + (2.4976E-004) * T^2 − (5.4164E-005) * S^2.

PEP = − 0.6887 + 0.1467 * T + 0.0919 * S − (3.2000E-003) * T * S − (7.5789E-004) * T^2 − (2.5789E-004) * S^2

TRYP = − 2854.8924 + 19.1371 * T + 247.3842 * S + 1.4045 * T * S − 1.0933 * T^2 – 5.0919 * S^2

GPX-hepatopancreas = + 16.8759 − 0.4343 * T − 0.8073 * S + (9.5500E-003) * T * S + (4.4360E-003) * T^2 + (9.9370E-003) * S^2.

SOD-hepatopancreas = + 133.7362 − 4.1737 * T − 5.3563 * S + 0.0722 * T * S + 0.0385 * T^2 + 0.0590 * S^2.

Variance analysis of digestive indexes and immune genes in hepatopancreas were presented in Tables 1. The results showed that the established models of salinity and temperature on digestive physiology of *P. fucata* were significant (*P* < 0.05), and the lack of fit was nonsignificant (*P* > 0.05), indicating that the regression model was significant. The fitting between experimental data and the model was good, and the model was suitable. The experimental results caused by unknown factors had little interference. The R^2^ of the established model was 0.9543, 0.9396, 0.9271, 0.9558, 0.9322 and 0.9246, respectively; the AdjR^2^ was 0.9335, 0.9122, 0.8940, 0.9357, 0.9014 and 0.8904, respectively; and the PredR^2^ was 0.8530, 0.8276, 0.7687, 0.8611, 0.7323 and 0.7083, respectively, so the model was appropriate.

**Table 1:** Model variance analysis of digestive indexes.

Response	Source	Quadratic sum	df	Mean square	F value	*P* value	
AMS	Model	0.760	5	0.150	45.91	< 0.0001	Significant
	Residual error	0.037	11	3.327E-003			
	Lack of fit	0.016	3	5.439E-003	2.15	0.1726	Not significant
	Pure error	0.020	8	2.535E-003			
	Total deviation	0.800	16				
LPS	Model	5.474E-003	5	1.095E-003	34.23	< 0.0001	Significant
	Residual error	3.518E-004	11	3.198E-005			
	Lack of fit	1.331E-004	3	4.436E-005	1.62	0.2594	Not significant
	Pure error	2.187E-004	8	2.734E-005			
	Total deviation	5.825E-003	16				
PEP	Model	0.100	5	0.021	28.00	< 0.0001	Significant
	Residual error	8.072E-003	11	7.338E-004			
	Lack of fit	2.702E-003	3	9.006E-004	1.34	0.3277	Not significant
	Pure error	5.370E-003	8	6.713E-004			
	Total deviation	0.110	16				
TRYP	Model	82,652.860	5	16,530.570	47.60	< 0.0001	Significant
	Residual error	3820.370	11	347.310			
	Lack of fit	1053.460	3	351.150	1.02	0.4351	Not significant
	Pure error	2766.910	8	345.860			
	Total deviation	86,473.230	16				
GPX	Model	1.190	5	0.240	30.26	< 0.0001	Significant
	Residual error	0.086	11	7.837E-003			
	Lack of fit	0.050	3	0.017	3.71	0.0614	Not significant
	Pure error	0.036	8	4.508E-003			
	Total deviation	1.270	16				
SOD	Model	49.010	5	9.800	26.99	< 0.0001	Significant
	Residual error	3.990	11	0.360			
	Lack of fit	1.890	3	0.630	2.39	0.1438	Not significant
	Pure error	2.100	8	0.260			
	Total deviation	53.000	16				

*AMS* Amylase activity in hepatopancreas, *LPS* Lipase activity in hepatopancreas, *PEP* Pepsin activity in hepatopancreas, *TRYP* Trypsin activity in the hepatopancreas, *GPX* Relative expression levels of GPX antioxidant genes in hepatopancreas, SOD Relative expression levels of SOD antioxidant genes in hepatopancreas.

The regression equations of salinity and temperature on the respiratory and metabolic physiological indexes of *P. fucata* (each coefficient is the actual value) established in the experiment were as follows:

LDH = + 6477.9344 − 235.5640 * T − 225.9151 * S + 1.6170 * T * S + 3.9368 * T^2 + 3.1262 * S^2

NKA = + 37.2763 − 0.7110 * T − 1.6076 * S + 0.0188 * T * S − (1.0868E -003) * T^2 + 0.0192 * S^2

AKP = + 2059.6119 − 109.0579 * T − 21.2943 * S − 1.7825 * T * S + 3.2400 * T^2 + 0.9106 * S^2

GPX-gill = + 13.5320 − 0.3348 * T − 0.6105 * S + (6.9700E-004) * T * S + (5.1916E − 003) * T^2 + 0.0111 * S^2.

SOD-gill = + 32.6290 − 1.5447 * T − 0.8288 * S + 0.0213 * T * S + 0.0175 * T^2 + (4.9017E-003) * S^2.

Variance analysis was performed on respiratory metabolism indexes and immune genes in gills (Table 2). The results showed that the established models of the effects of salinity and temperature on respiratory metabolism physiology of *P. fucata* were significant (*P* < 0.05), and the lack of fit was nonsignificant (*P* > 0.05), indicating that the regression model was significant, and the fitting between the experimental data and the model was good, the model was suitable, and the experimental results caused by unknown factors had little interference. The R^2^ of the established model was 0.9587, 0.9699, 0.9358, 0.9492 and 0.9294, respectively; the AdjR^2^ was 0.9400, 0.9562, 0.9066, 0.9262 and 0.8973, respectively; and the PredR^2^ was 0.8469, 0.8730, 0.7314, 0.8614 and 0.7794, respectively, indicating that the model was appropriate.

**Table 2:** Model variance analysis of respiratory metabolic indicators.

Response	Source	Quadratic sum	df	Mean square	F value	*P* value	
LDH	Model	1.074E + 005	5	21,473.120	51.10	< 0.0001	Significant
	Residual error	4622.790	11	420.250			
	Lack of fit	2668.480	3	889.490	3.64	0.0639	Not significant
	Pure error	1954.310	8	244.290			
	Total deviation	1.120E + 005	16				
NKA	Model	14.200	5	2.840	70.87	< 0.0001	Significant
	Residual error	0.440	11	0.040			
	Lack of fit	0.260	3	0.088	3.95	0.0533	Not significant
	Pure error	0.180	8	0.022			
	Total deviation	14.640	16				
AKP	Model	84,824.460	5	16,964.890	32.07	< 0.0001	Significant
	Residual error	5818.580	11	528.960			
	Lack of fit	3318.580	3	1106.190	3.54	0.0678	Not significant
	Pure error	2500.000	8	312.500			
	Total deviation	90,643.040	16				
GPX	Model	1.190	5	0.240	41.13	< 0.0001	Significant
	Residual error	0.064	11	5.777E-003			
	Lack of fit	0.018	3	5.848E-003	1.02	0.4345	Not significant
	Pure error	0.046	8	5.750E-003			
	Total deviation	1.250	16				
SOD	Model	3.150	5	0.630	28.97	< 0.0001	Significant
	Residual error	0.240	11	0.022			
	Lack of fit	0.088	3	0.029	1.54	0.2768	Not significant
	Pure error	0.150	8	0.019			
	Total deviation	3.390	16				

*LDH* Lactate dehydrogenase activity in gills, *NKA* Na^+^-K^+^-ATPase activity in gills, *AKP* Alkaline phosphatase activity in gills, *GPX* Relative expression levels of GPX antioxidant genes in gills, *SOD* Relative expression levels of SOD antioxidant genes in gills.

### Model coefficient estimation

The established regression model of digestive and respiratory metabolism indexes were estimated by coefficients, and the results were shown in Table 3 and Table 4. The coefficients in Table 3 and Table 4 were coded values (elimination of units among coefficients), and their effects were directly reflected by numerical values. 95% confidence interval (C.I.) explained the change of coefficient coding value in 95% interval.

**Table 3:** Coefficient estimation of the digestive index prediction model equation.

Response	Factor	Coefficient estimation	*P* value	Standard error	95% C.I	
					Low	High
AMS	Intercept	1.100	–	0.023	1.050	1.150
	T	0.067	0.0070	0.020	0.023	0.110
	S	0.055	0.0208	0.020	0.010	0.100
	T*S	−0.25	< 0.0001	0.029	−0.310	−0.180
	T^2^	−0.20	< 0.0001	0.028	−0.260	−0.130
	S^2^	−0.26	< 0.0001	0.028	−0.320	−0.190
LPS	Intercept	2.130	–	2.247E-003	2.120	2.130
	T	0.025	< 0.0001	1.999E-003	0.020	0.029
	S	6.940E-003	0.0052	1.999E-003	2.539E-003	0.011
	T*S	−6.570E-004	0.8205	2.828E-003	−6.880E-003	5.566E-003
	T^2^	6.244E-003	0.0444	2.752E-003	1.865E-004	0.012
	S^2^	−1.354E-003	0.6324	2.752E-003	−7.412E-003	4.703E-003
PEP	Intercept	2.640	–	0.011	2.610	2.660
	T	0.096	< 0.0001	9.577E-003	0.075	0.120
	S	−0.012	0.2185	9.577E-003	−0.034	8.579E-003
	T*S	−0.080	0.0001	0.014	−0.110	−0.050
	T^2^	−0.019	0.1785	0.013	−0.048	0.010
	S^2^	−6.447E-003	0.6344	0.013	−0.035	0.023
TRYP	Intercept	858.150	–	7.410	841.850	874.450
	T	19.000	0.0149	6.590	4.500	33.500
	S	−13.230	0.0698	6.590	−27.740	1.270
	T*S	35.110	0.0031	9.320	14.600	55.620
	T^2^	−27.330	0.0118	9.070	−47.290	−7.370
	S^2^	−127.300	< 0.0001	9.070	−147.260	−107.330
GPX	Intercept	0.660	–	0.035	0.580	0.740
	T	0.270	< 0.0001	0.031	0.210	0.340
	S	−0.060	0.0799	0.031	−0.130	8.515E-003
	T*S	0.240	0.0002	0.044	0.140	0.340
	T^2^	0.110	0.0259	0.043	0.016	0.210
	S^2^	0.250	0.0001	0.043	0.150	0.340
SOD	Intercept	0.330	–	0.240	−0.190	0.860
	T	−1.120	0.0003	0.210	−1.590	−0.650
	S	−1.230	0.0001	0.210	−1.700	−0.760
	T*S	1.810	< 0.0001	0.300	1.140	2.470
	T^2^	0.960	0.0073	0.290	0.320	1.610
	S^2^	1.480	0.0004	0.290	0.830	2.120

**Table 4:** Coefficient estimation of the respiratory metabolic index prediction model equation.

Response	Factor	Coefficient estimation	*P* value	Standard error	95% C.I	
					Low	High
LDH	Intercept	306.540	–	8.150	288.610	324.470
	T	32.760	0.0009	7.250	16.810	48.710
	S	−52.120	< 0.0001	7.250	−68.070	−36.170
	T*S	40.420	0.0023	10.250	17.860	62.990
	T^2^	98.420	< 0.0001	9.980	76.460	120.380
	S^2^	78.150	< 0.0001	9.980	56.200	100.110
NKA	Intercept	2.000	–	0.080	1.820	2.170
	T	−1.200	< 0.0001	0.071	−1.360	−1.050
	S	−0.310	0.0010	0.071	−0.470	−0.160
	T*S	0.470	0.0007	0.100	0.250	0.690
	T^2^	−0.027	0.7855	0.097	−0.240	0.190
	S^2^	0.480	0.0004	0.097	0.270	0.690
AKP	Intercept	228.030	–	9.140	207.920	248.150
	T	15.150	0.0893	8.130	−2.750	33.050
	S	−74.330	< 0.0001	8.130	−92.220	−56.430
	T*S	−44.560	0.0026	11.500	−69.870	−19.250
	T^2^	81.000	< 0.0001	11.190	56.360	105.630
	S^2^	22.760	0.0668	11.190	−1.870	47.400
GPX	Intercept	0.490	–	0.030	0.430	0.560
	T	−0.280	< 0.0001	0.027	−0.340	−0.220
	S	0.140	0.0003	0.027	0.080	0.200
	T*S	0.017	0.6555	0.038	−0.066	0.100
	T^2^	0.130	0.0049	0.037	0.048	0.210
	S^2^	0.280	< 0.0001	0.037	0.200	0.360
SOD	Intercept	0.520	–	0.059	0.400	0.650
	T	−0.360	< 0.0001	0.052	−0.470	−0.240
	S	−0.110	0.0638	0.052	−0.220	7.328E-003
	T*S	0.530	< 0.0001	0.074	0.370	0.700
	T^2^	0.440	< 0.0001	0.072	0.280	0.600
	S^2^	0.120	0.1158	0.072	−0.035	0.280

Table 3 showed that the minimum values of 95% C.I. prediction of digestive index intercept were 1.05, 2.12, 2.61, 841.85, 0.58 and -0.19, respectively, and the maximum values were 1.15, 2.13, 2.66, 874.45, 0.74 and 0.86, respectively.

The test and analysis results of the model coefficients showed that the primary and secondary effects of salinity and temperature and their interaction effects had significant indigenous effects on AMS (*P* < 0.05). The primary and secondary effects of salinity and temperature had significant effects on LPS (*P* < 0.05), while the secondary effects of salinity and their interaction with temperature had no significant effects on LPS (*P* > 0.05). The primary effect of temperature and the interaction effect of temperature and salinity had significant influence on PEP (*P* < 0.05), while the primary effect of salinity and the secondary effect of temperature and salinity had no significant influence on PEP (*P* > 0.05). The primary, secondary and interactive effects of salinity and temperature had significant effects on TRYP (*P* < 0.05). The primary and secondary effects of temperature, the secondary effects of salinity and the interaction between temperature and salinity had significant effects on the relative gene expression of GPX in hepatopancreas (*P* < 0.05), while the primary effect of salinity had no significant effect on the relative gene expression of GPX in hepatopancreas (*P* > 0.05). The primary, secondary and interaction effects of temperature and salinity had significant effects on the relative gene expression of SOD in hepatopancreas (*P* < 0.05).

Table 4 showed that the minimum values predicted by the 95% C.I. of the intercept of respiratory metabolism indexes were 288.61, 1.82, 207.92, 0.43 and 0.40, respectively, and the maximum values were 324.47, 2.17, 248.15, 0.56 and 0.65, respectively. The test results of the model coefficients showed that the primary, secondary and interaction effects of salinity and temperature had significant effects on LDH (*P* < 0.05). Primary effect of salinity and temperature and their interaction effect, secondary effect of salinity had significant indigenous effect on NKA (*P* < 0.05), secondary effect of temperature had no significant indigenous effect on NKA (*P* > 0.05). The primary effect of temperature and the secondary effect of salinity had no significant effect on AKP (*P* > 0.05), while the primary effect of salinity, the secondary effect of temperature and the interaction between temperature and salinity had significant indigenous effect on AKP (*P* < 0.05). The primary and secondary effects of salinity and temperature had significant effects on the relative expression of GPX gene in gills (*P* < 0.05), and the interaction of salinity and temperature had no significant effect on the relative expression of GPX gene in gills (*P* > 0.05). The primary and secondary effects of temperature and the interaction of salinity and temperature had significant effects on the relative expression of SOD gene in gills (*P* < 0.05), while the primary and secondary effects of salinity had no significant effects on the relative expression of SOD gene in gills (*P* > 0.05).

### Response surface analysis of digestive and respiratory metabolism

**Figure 1:**
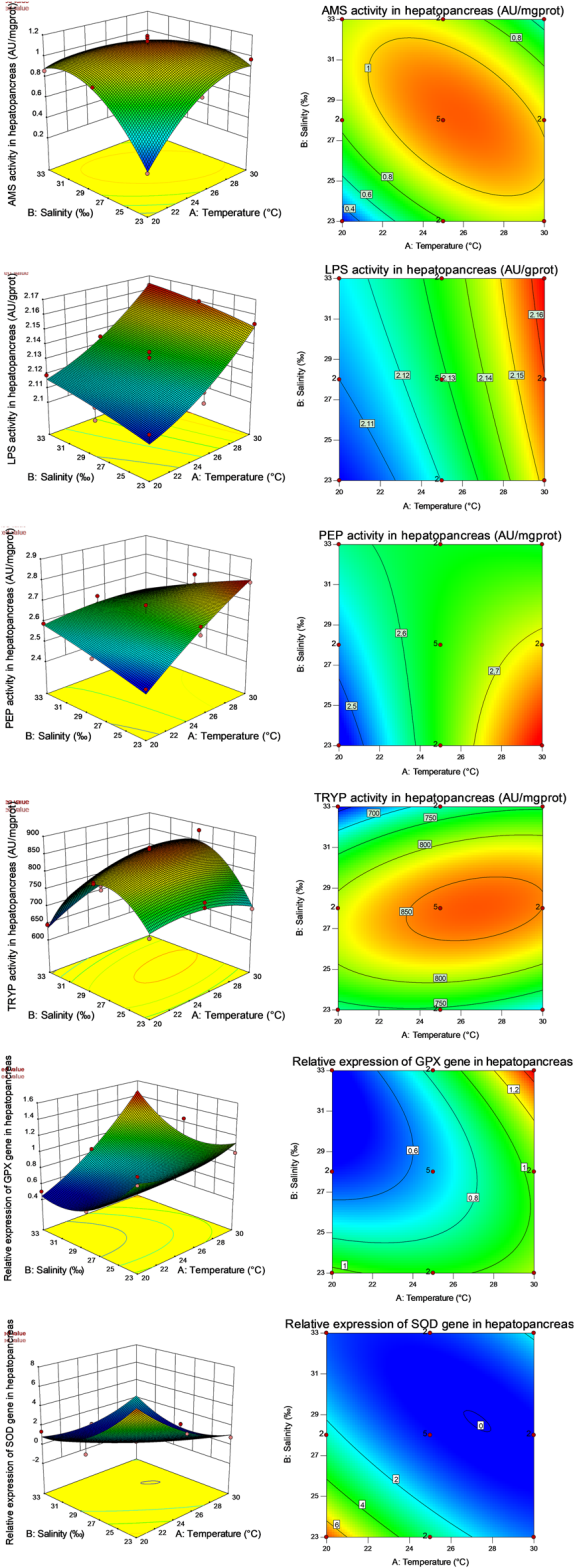
Response surface plots (left) and contour plots (right) of salinity and temperature to digestive enzyme activities and hepatopancreas immune gene relative expression of *P. fucata*. The activities of AMS, LPS, PEP and TRYP, as well as the relative expression levels of GPX and SOD genes in hepatopancreas were shown from top to bottom. The heat map in Fig. 1 was generated using Design Expert (https://www.statease.com/software/design-expert/, version 10.0.7.0 32-bit) software.

Under all different combinations of salinity and temperature, the digestive enzyme activity and the relative expression of immune genes in hepatopancreas tissue estimated according to the fitted response surface were shown in Fig. 1. By examining the response surface or contour, it was found that the relative expression levels of AMS, TRYP and hepatopancreas SOD genes were unimodal. AMS and TRYP increased at first and then decreased with increasing temperature or salinity, while the relative expression of SOD gene in hepatopancreas decreased at first and then increased with increasing temperature or salinity. LPS increased with increasing two factors. PEP increased with increasing temperature and decreased with increasing salinity. The relative expression level of hepatopancreas GPX gene showed an upward trend with increasing temperature, and decreased at first and then increased with increasing salinity.

**Figure 2:**
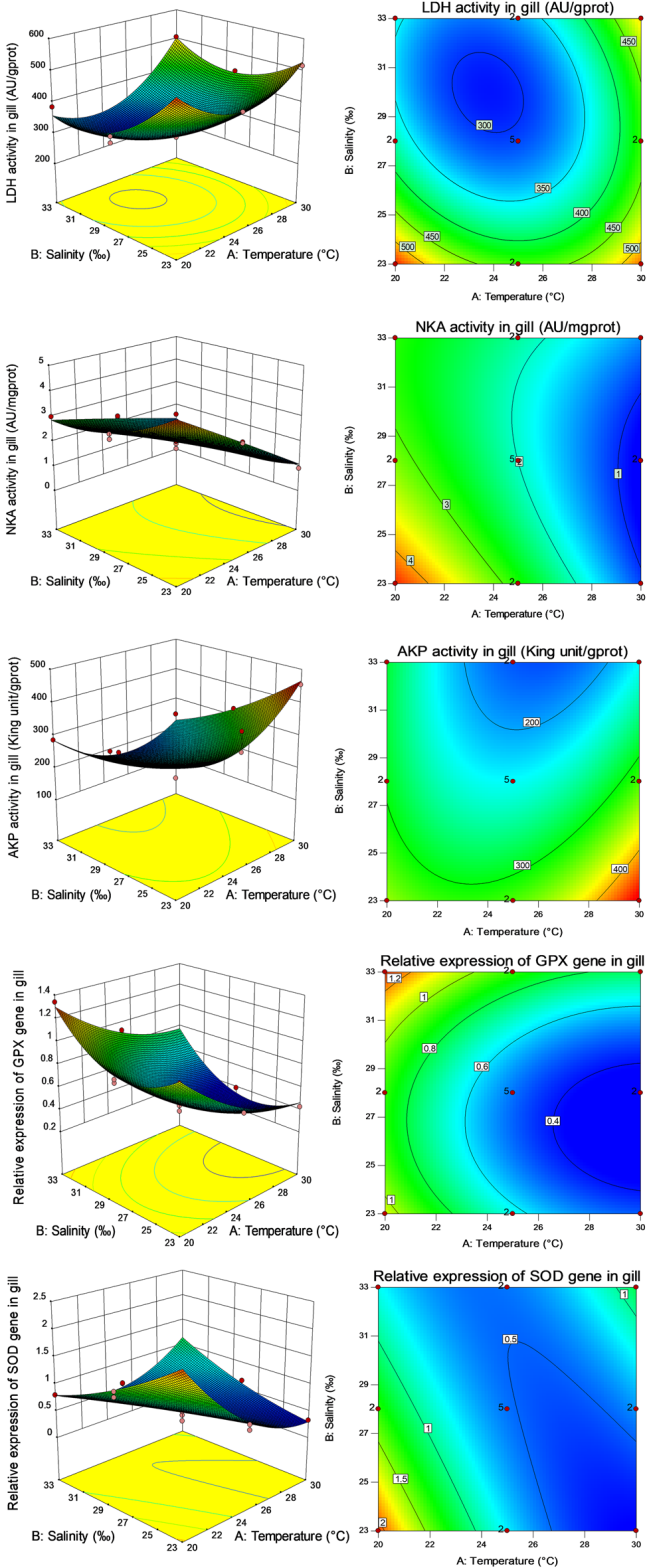
Response surface (left) and contour map (right) of salinity and temperature to respiratory metabolic enzyme activities and gill immune gene relative expression of *P. fucata*. The activities of LDH, NKA and AKP, and the relative expression levels of GPX and SOD genes in gills were shown from top to bottom. The heat map in Fig. 2 was generated using Design Expert (https://www.statease.com/software/design-expert/, version 10.0.7.0 32-bit) software.

Under all different combinations of salinity and temperature, the activities of respiratory metabolic enzymes and the relative expression of immune genes in gills estimated by the fitted response surface were shown in Fig. 2. By examining the response surface or contour, it was found that LDH was a single peak, and its activity showed a U-shaped trend with the increase in salinity or temperature. NKA showed a decreasing trend with the increase in temperature, and a U-shaped trend with the increase in salinity. AKP showed a U-shaped trend with the increase in temperature, and decreased with the increase in salinity. The relative expression of GPX gene in gills showed a decreasing trend with the increase in temperature, and a U-shaped trend with the increase in salinity. The relative expression of SOD gene in gills showed a U-shaped trend with the increase in temperature, and decreased with the increase in salinity.

### Optimization

The results showed that the optimal combination of temperature and salinity was 26.288 °C/28.272 ‰, and the maximum value of AMS, LPS, PEP and TRYP was 1.107, 2.136, 2.660 and 860.627, respectively. The minimum relative expression levels of GPX and SOD genes in hepatopancreas were 0.740 and 0.070, respectively. The lowest relative expression levels of GPX and SOD genes in gills were 0.439 and 0.463, respectively, and the lowest values of LDH, NKA and AKP were 319.477, 1.677 and 232.716, respectively. The desirability was 0.832.

## Discussion

Temperature and salinity are the key factors affecting the digestive enzyme activity of shellfish^41^. As an osmotic animal, shellfish will actively discharge salt or water to adapt to environmental changes when the environmental salinity is too low or too high^42^. In the process of osmotic pressure regulation, the digestive ability of shellfish decreases with the consumption of large amounts of energy^43^. The results of the present study showed that the digestive enzyme activity of *P. fucata* was relatively high at the salinity of 28 ‰, and the activities of AMS and TRYP decreased when the salinity was low or high, which was consistent with the above laws and consistent with the results of juvenile scallop *Chlamys nobilis*^44^ and *Solen grandis*^43^. There are few reports on the effect of salinity on digestive enzyme activity in shellfish, except for those consistent with the above view^45,46^, Chiu and Benitez^47^ also pointed out that inorganic ions in seawater can be used as activators of digestive enzymes at appropriate concentrations, and inhibitors below the appropriate range.

According to the kinetics of enzymatic reaction and the protein properties of digestive enzymes, the speed of enzymatic reaction is accelerated with the increase in temperature in a certain temperature range, and begins to decrease beyond a certain temperature range. As a thermophilic animal, the digestive enzyme activity of shellfish is directly affected by environmental temperature changes. Digestive enzymes need optimal temperature to better participate in the biological reaction process^44^. The present study showed that when temperature was about 26 °C, digestive enzymes reached the maximum activity value, and AMS and TRYP showed an inverted U-shaped trend with the increase in temperature, which was in line with the above laws and was consistent with *Haliotis diversicolor*^48^, *Chlamys nobilis*^44^, *Chlamys farreri*^49^, *Lutraria sieboldii* Reeve^50^, *Solen grandis*^43^, and *Pinctada martensii*^51^ had the same results. The increase of LPS with temperature or salinity may be due to the low-fat intake in shellfish diet and the fluctuation of LPS in the lower activity range. Different digestive enzyme activities in vivo are related to different feeding habits of different shellfish. Bivalves such as *Mytilus edulis* have higher protease and AMS activities due to their preference for unicellular algae, while gastropods such as *Littorina sp.* have higher cellulase activities due to their preference for macroalgae^52^. The inconsistency may be related to the difference in enzyme activity determination methods, species, age, feeding habits and breeding conditions.

Results from the present study showed that the primary and secondary effects of temperature and salinity had significant indigenous effects on the AMS of *P. fucata*, indicating that the AMS activity was susceptible to temperature and salinity, and it was in line with the above law of digestive enzyme activity changing with temperature and salinity. This may be because the salinity changes affected the osmotic pressure of the body, and AMS was a biological macromolecule. The enzyme activity was affected by the increasing of temperature, and high temperature may even caused denaturation (inactivation). Therefore, the maximum value of AMS will only reach at the optimum temperature and salinity^46,53^. Incidentally, the peak value here was the optimum temperature of digestive enzyme activity in *P. fucata*. In the study of *Chlamys farreri*^49^, *Haliotis discus hannai* Ino^41^ and *Sinonovacula constricta*^54^, it was found that the suitable temperature for the growth of shellfish was lower than the temperature of its main digestive enzyme activity. The optimal temperature obtained in the present study was 26 °C, which was close to the result of 27 °C of juvenile *P. martensii* by Zhu^55^. This may be because the present study and Zhu^55^ experiment were the activity of digestive enzymes secreted by shellfish stimulated by ambient temperature, and other studies were environmental stimulation enzyme solution. The primary effect of salinity on TRYP was not significant, but the secondary effect was significant, and the primary effect of temperature on PEP was significant, but the secondary effect was not significant, indicating that TRYP activity was nonlinear with salinity, and PEP was linear with temperature. This may be because interaction between factors shields one or two effects^44,55^.

Previous studies mostly focused on the effects of single factor (factor) on digestive enzyme activity in aquatic organisms, but few on the interaction between factors. In the case of interaction between factors, the investigation of interaction between factors is much more important than the investigation of the main effect (primary or secondary effect) of a single factor^15^. In the present study, Box-Benhnken design (BBD) was used to conduct the significant analysis and test of the interaction between salinity and temperature. The results showed that the combined effect of salinity and temperature had significant influence on the activities of AMS, PEP and TRYP. It may be because the superposition of two main effects (salinity and temperature) has an impact on digestive enzyme activity, which was similar to the results reported by Zhu^55^ and Qian, et al.^44^. However, in this study, the interaction between salinity and temperature on LPS in *P. fucata* was not significant, indicating that the effects of salinity and temperature on LPS were independent rather than antagonistic or synergistic, or the two effects (salinity and temperature) were mutually shielded, resulting in the weakening of the interaction between the two^44^, or the influence mechanisms of salinity and temperature on the enzyme activity of *P. fucata* were different. There were also no significant results of interaction between reproduction and embryonic development of *P. fucata*^1^, *R. cuneata*^21^, *P. margaritifera*^56^ and noble scallop *Chlamys nobilis*^26^. Such variations of salinity and temperature may due to the species difference as different species have different osmoregulation modes and effects.

As an important part of energy metabolism research, respiratory metabolism is an important presentation and expression of metabolic activities in aquatic animals. Oxygen, metabolic level and physiological status required for maintaining the lowest metabolic level of aquatic animals can be directly or indirectly reflected by it^57^. During the life activities of shellfish, respiratory metabolism reflects the physiological status, metabolic characteristics and adaptability to external environmental stress^28^. Salinity and temperature are important environmental factors affecting the respiratory metabolism of aquatic organisms. Salinity can affect the regulation of osmotic pressure to affect the metabolism of the body, while temperature can affect biological oxygen consumption and osmotic pressure and ion regulation to affect the metabolism level^57^.

NKA is a kind of P-type ATPase, which can actively transport Na^+^ out of cells and K^+^ into cells. It is essential to maintain cell osmotic pressure^28^. Its activity provides a major driving force for activating other ion transport systems involved in osmotic regulation^58^. In this study, NKA showed a U-shaped distribution with the increase in salinity, which was consistent with the results of black and red shell *P. fucata* after 1.5 h salinity stress^28^ and red shell *P. fucata* after 12 h salinity stress^29^ . It indicated that *P. fucata* had strong adaptability to low or high salt. Different from the results of *Meretrix lusoria*^58^ and *Pomacea canaliculata*^59^, salinity has different effects on respiration metabolism in different species. In the primary and secondary effects, the effect of salinity on NKA of *P. fucata* was significant, indicating that NKA was easily affected by salinity changes, and showed a U-shaped trend with the increase in salinity, which might be due to the change of osmotic pressure caused by salinity changes, and the enhancement of NKA activity to maintain the osmotic pressure balance in the body^44^. In this study, the interaction between temperature and salinity had a significant indigenous effect on NKA in *P. fucata*, while in the study of *Chlamys nobilis*^44^, the effect on NKA activity was not significant, which was different from the results of this study, one may be superimposed by the main effect, while the other was shielded from each other. This may be due to different species and different osmotic pressure regulation modes, so that the degree of NKA on the membrane was different under the interaction (synergistic effect) of salinity and temperature. Osmotic regulation of *P. fucata* is not entirely achieved by NKA enzyme in gills^28^.

AKP can catalyze the transfer reaction of phosphate groups and the hydrolysis of phosphate monolipids. As an important enzyme for the survival and growth of aquatic organisms, AKP also helps the body to form and secrete chitin, absorb calcium in water and form calcium phosphate^60^. The present study showed that AKP showed a U-shaped distribution with the increase in temperature, indicating that the increase of temperature accelerated the respiratory metabolism level of *P. fucata* to a certain extent and increased the ATP production to support the continuous movement of *P. fucata*. Contrary to the findings of *Clinocardium californiense*^60^. Evidence indicates that *Clinocardium californiense* is not tolerant to high temperature, while *P. fucata* has strong adaptability to high temperature and low temperature. Results from the present study showed that the secondary effect of temperature had a significant indigenous effect on AKP, indicating that there was a peak value of AKP within the set temperature range. The response surface diagram also clearly showed that there was a minimum value of AKP with the increase in temperature. This may be because *P. fucata* begins to secrete large amounts of AKP at lower or higher temperatures to resist adverse environments in order to regulate immunity^61^. The interaction between salinity and temperature had significant effects on AKP in *P. fucata*, indicating that osmotic pressure regulation and temperature were closely related to respiratory metabolism in *P. fucata*. This may be due to the fact that salinity provides metal ions to activate enzymes, and temperature changes the conformation of AKP, so that the two can jointly promote the regulation of enzyme activity in the process of substrate-phosphatase binding and activation^62^.

LDH is an important glycolytic enzyme required for cell energy metabolism, which can convert lactic acid, the main by-product of anaerobic glycolysis, into pyruvic acid and release energy. LDH plays a key role in maintaining aerobic metabolism, and its activity is closely related to cell metabolism, which can reflect the level of anaerobic respiration to a certain extent^28^. In the present study, when the temperature and salinity changed from 20 to 30 °C and from 23 to 33 ‰, respectively, the LDH activity decreased at first and then increased, indicating that the anaerobic respiration level of *P. fucata* presented a U-shaped distribution with the increase in temperature or salinity. In the study of *C. californiense*^60^, it was found that LDH activity increased with the increasing in temperature in the range of 16–28 °C. In the present study, the LDH activity of *P. fucata* was consistent with its law at 26–30 °C. When temperature was about 26 °C, and the salinity was about 28‰, the LDH activity was the lowest, indicating that the salinity and temperature were appropriate at this time, and the anaerobic metabolism level was the lowest in the body. It was speculated that aerobic metabolism was the main part at this time, and a large amount of energy generated could be used for the body to cope with environmental changes.

The interaction between marine invertebrate environmental factors and antioxidant enzymes has been intensively studied. Mostly reflected in the stress of various factors, enzyme activity determination and expression level of space–time, tissue differences. There are still many studies on the genes of antioxidant enzymes related to stress resistance in marine organisms, such as GPX, SOD and GST^63^. However, there are few studies on the effects of temperature or salinity on the relative expression of SOD and GPX genes in shellfish. In this study, the primary and secondary effects of temperature had significant effects on GPX and SOD gene expression in hepatopancreas and gill, indicating that they were nonlinear with temperature, and temperature had significant effects. Among them, the relative expression of hepatopancreas SOD and gill SOD had the minimum value. The expression of GPX in hepatopancreas was up-regulated with the increase in temperature, while the expression of GPX in gills was just the opposite. This may be because the gene expression of GPX in gills affected by temperature is lagging behind that in hepatopancreas, and also showed the tissue specificity of gene expression.

In this study, the primary and secondary effects of salinity have significant effects on hepatopancreas SOD gene expression and gill GPX gene expression, indicating that salinity has a significant effect on it and a nonlinear relationship. The secondary effect of salinity on the relative expression of SOD in gills was shielded by the interaction of temperature and salinity. The secondary effect of salinity had a significant impact on the relative expression of GPX and SOD genes in hepatopancreas and GPX genes in gills of *P. fucata*. It can be seen that they have the optimal value within the specified range. At this time, the environment was suitable for *P. fucata*, and the relative expression of antioxidant genes was down-regulated, which indirectly supported the above results on digestive enzymes and respiratory metabolic enzymes.

## Conclusion

In summary, environmental salinity and temperature changes may regulate the growth, energy utilization and metabolism of *P. fucata* by affecting digestive and respiratory metabolism and antioxidant capacity of hepatopancreas and gill. Our study showed that the combined effect of salinity and temperature had a significant indigenous effect on the digestion and respiratory metabolism of *P. fucata*. Therefore, the effects of single factor and synergistic effect should be considered in the artificial breeding and breeding of *P. fucata*. Results from the present study provide a theoretical basis for future research and digestive enzyme model establishment.

## Materials and methods

### Experimental animals

The pearl oysters (total weight: 34.19 ± 1.39 g, shell length: 50.75 ± 1.43 mm) were collected from South China Sea, and then transferred to the laboratory of Lingshui Station (Hainan, China) for acclimation. They were conditioned in some 5000 L cement tanks in the laboratory at ambient temperature (25 ± 1 °C) and salinity (33 ± 1 ‰) prior to experiment. During one week of acclimation, ambient parameter remained DO > 6.5 mg / L, pH 8.0 ± 0.1, light intensity < 500 Lx with natural photoperiod and *Platymonas subcordiformis* (200 × 10^3^ cells/mL) was fed once a day at regular intervals (9: 00–9: 30 a.m.). Half of the seawater was replaced and feces, residues were siphoned off daily, and dead pearl oysters were removed from the tank immediately.

### Experimental design

The respiratory metabolism and digestion were assessed utilizing the Box-Benhnken design (BBD) with either 2-factors or explanatory variables, salinity (S, ‰) and temperature (T, °C). Each factor contained 3-levels, which were coded as − 1, 0 and 1, respectively (Table 5). There were nine salinity–temperature combined treatments in the experiment, each treatment had three replicates, and each replicate contained 15 *P. fucata* (Table 6, Table 7). The experiment was carried out in twenty-seven 800-L cement tanks.

**Table 5:** Factors and levels table of Box-Benhnken.

Factor	Level		
	− 1	0	1
S (‰)	23	28	33
T (°C)	20	25	30

**Table 6:** Experimental design and results of digestive response surface.

Run	Coded		Actual		AMS	LPS	PEP	TRYP	GPX-hepatopancreas	SOD-hepatopancreas
	T(℃)	S (‰)	T(℃)	S (‰)	(AU/mgprot)	(AU/gprot)	(AU/mgprot)	(AU/mgprot)	–	–
1	0	1	25	33	0.89	2.132	2.61	712.33	0.853	0.5369
2	−1	0	20	28	0.86	2.101	2.51	815.34	0.483	2.8700
3	0	0	25	28	1.19	2.131	2.62	849.45	0.612	0.4369
4	−1	−1	20	23	0.27	2.107	2.47	719.76	1.020	7.5500
5	0	1	25	33	0.89	2.135	2.65	699.35	0.833	0.5030
6	0	0	25	28	1.02	2.120	2.63	855.48	0.632	0.4769
7	1	−1	30	23	0.97	2.154	2.79	692.74	0.993	0.7753
8	−1	0	20	28	0.86	2.110	2.51	812.22	0.483	0.8277
9	0	−1	25	23	0.77	2.120	2.62	747.35	1.075	2.8700
10	0	0	25	28	1.14	2.131	2.63	864.28	0.603	0.4469
11	1	0	30	28	0.92	2.160	2.76	812.35	1.009	0.5300
12	0	0	25	28	1.17	2.135	2.68	868.34	0.632	0.3069
13	1	1	30	33	0.58	2.163	2.59	758.78	1.431	1.8300
14	0	−1	25	23	0.77	2.113	2.66	761.84	1.015	2.8700
15	−1	1	20	33	0.86	2.119	2.59	645.35	0.503	1.3800
16	0	0	25	28	1.07	2.135	2.62	855.74	0.692	0.4469
17	1	0	30	28	0.92	2.159	2.71	880.82	1.251	0.5030

**Table 7:** Experimental design and results of respiratory metabolic response surface.

Run	Coded		Actual		LDH	NKA	AKP	GPX-gill	SOD-gill
	T (℃)	S (‰)	T (℃)	S (‰)	(AU/gprot)	(AU/mgprot)	(King unit /gprot)	–	–
1	0	1	25	33	317.87	2.26	151.31	0.901	0.6010
2	−1	0	20	28	341.31	3.12	319.61	0.840	1.1900
3	0	0	25	28	322.16	1.92	223.45	0.565	0.4219
4	−1	−1	20	23	556.99	4.61	314.79	1.110	2.2400
5	0	1	25	33	317.87	2.16	181.31	0.930	0.6100
6	0	0	25	28	300.68	2.02	223.45	0.385	0.7819
7	1	−1	30	23	515.69	0.92	454.22	0.426	0.3266
8	−1	0	20	28	361.31	2.92	299.61	0.870	1.2900
9	0	−1	25	23	435.70	2.77	316.74	0.661	0.6130
10	0	0	25	28	287.16	2.22	223.45	0.435	0.4919
11	1	0	30	28	442.79	1.07	324.91	0.391	0.6600
12	0	0	25	28	312.16	2.01	223.45	0.445	0.3119
13	1	1	30	33	505.90	1.18	248.22	0.726	1.0200
14	0	−1	25	23	435.70	2.82	376.74	0.591	0.7130
15	−1	1	20	33	385.50	3.00	287.04	1.340	0.8022
16	0	0	25	28	342.16	1.72	223.45	0.635	0.6619
17	1	0	30	28	442.79	0.87	314.91	0.391	0.6600

Throughout the experiment, salinity ranged between 23 and 33 ‰, and the minimum temperature was set at 20 °C, the maximum at 30 °C. Preliminary trials, based upon the seasonal changes in salinity and temperature of the seawater in the South China Sea, were conducted to define the settings of salinity and temperature chosen for the combination experiment. Temperatures for the respiratory metabolism and digestion experiments were manipulated by heating rod or ice bottles. All salinities were prepared through addition of sea salts or tap water with 24 h aeration to the natural double-filtered seawater, and a salinometer (ATAGO S-10E) was used to gauge the salinity.

#### Collection and determination of tissue samples

Three shellfish were randomly collected from each parallel, and the hepatopancreas and gill tissues were cut off on an ice tray with scissors. Rinsed with pre-cooling 0.9% normal saline, blotted with clean filter paper, the tissue samples were quickly placed in 2 mL centrifuge tubes and stored at − 80 °C. The biochemical parameters of gills and hepatopancreas tissue were determined according to the instructions of the manufacturer (Nanjing Jiancheng Institute of Biological Engineering, Nanjing, China) i.e., **hepatopancreas tissue:** amylase (AMS) (Item No. C016-1-1): starch-iodine colorimetric method; lipase (LPS) (Item No. A054-2-1): methyl halal substrate method (microplate method); trypsin (TRYP) (Item No. A080-2-2): ultraviolet colorimetric method; pepsin (PEP) (Item No. A080-1-1): colorimetric method; the total protein (TP) (Item No. A045-4-2): bicinchoninic acid (BCA) method; **gill tissue:** lactate dehydrogenase (LDH) (Item No. A020-2-2): 2,4-Dinitrophenylhydrazine method; Na^+^–K^+^–ATPase (NKA) (Item No. A070-2-2): inorganic phosphorus method; alkaline phosphatase (AKP) (Item No. A059-2-2): disodium diphenyl phosphate colorimetric method and the total protein (TP) (Item No. A045-4-2): bicinchoninic acid (BCA) method.

According to the kit manufacturer's instructions, regarding the use of arbitrary unit (AU) and King unit as units of enzyme activity in this study, the specific meaning of these units in each enzyme is as follows: For AMS, one unit of amylase activity (AU/mgprot) was defined as the hydrolysis of 10 mg of starch per mg of protein in tissue treated with substrate at 37 °C for 30 min. For LPS, each gram of tissue protein reacted with the substrate in this reaction system for 1 min at 37 °C, and each 1 μmol of substrate consumed was one unit of enzyme activity (AU/gprot). For PEP, 1 μg of tyrosine per milligram of tissue protein at 37 °C per minute was equivalent to one unit of enzyme activity (AU/mgprot). For TRYP, at pH 8.0, 37 °C, each milligram of trypsin contained in the protein changes the absorbance by 0.003 per minute, which was one unit of enzyme activity (AU/mgprot). For LDH, each gram of tissue protein was treated with substrate at 37 °C for 15 min, and 1 µmol pyruvate was produced in the reaction system as one unit (AU/gprot). For NKA, the amount of ATP breakdown to produce 1 µmol of inorganic phosphorus per milligram of tissue protein per hour was specified as one unit of atpase activity (AU/mgprot). For AKP, 1 mg of phenol per gram of tissue protein produced by interaction with substrate at 37 °C for 15 min was defined as 1 King unit/gprot.

### Primer design and gene expression

Genes selected and primers designed (Table 8) for qPCR analysis were conducted based on Adzigbli, et al.^64^ and Gu, et al^65^. RNA was extracted according to the method of Fu, et al.^66^. The ND 5000 spectrophotometer (BioTeke Corporation, China) and 1% agarose gel electrophoresis were used to evaluate the quantity and integrity of isolated RNA, respectively. Finally, the reverse transcription and relative gene expression were determined according to the method of Yang, et al.^67^.

**Table 8:** Sequences of the qPCR primers used in the study.

Gene	Primers	Sequences (5′-3′)
SOD	SOD F	TCCACCTGTCTGGGTTTGATGT
	SOD R	CCGGAGCACCAT GATTGACTTT
GPX	GPX F	GCTTGTCATTCTCGGTTTCC
	GPX R	TCAGGCTGGTAGATTCGTCA
β-Actin	β-Actin F	CGGTACCACCATGTTCTCAG
	β-Actin R	GACCGGATTCATCGTATTCC

### Statistical analysis

Box-Benhnken design (BBD) was used in the experiment, namely the specific combination of salinity and temperature at different levels. According to Ryan and Morgan^68^ and Montgomery^15^, this experimental design could more accurately estimate the influence of different factors (salinity and temperature in the present study), and expand the range of validity of the conclusion when additional factors are inserted. The relationship between quantitative correlation factors and response largely depended on the response surface of fitting experimental data. Therefore, the three-dimensional response surface map could be obtained by drawing and processing the response of different factors (here is salinity and temperature). Image rendering and data analysis were performed using Design Expert 10 (32-bit). Assuming that the response surface properties are described by the following models:

$$Y = \beta_{0} + \beta_{1} S + \beta_{2} T + \beta_{12} S \times T + \beta_{11} S^{2} + \beta_{22} T^{2} + \varepsilon$$

In the formula, Y was the response (digestive and respiratory metabolism related indicators), β_0_ was a constant, β_1_ represented the linear effect of salinity, β_2_ represented the linear effect of temperature, β_12_ represented the interaction effect of salinity and temperature; β_11_ was the secondary effect of salinity, β_22_ was the secondary effect of temperature, ε was the random error, the mean was zero (in line with normal distribution). The regression coefficients of these experimental points were calculated by the least square method. *P* < 0.05 of the items included in the above model was considered statistically significant or significant. The adequacy and importance of establishing the model could be proved by the generated variance analysis table. The fitting of the above model was expressed by the determination coefficient R^2^, and its statistical significance was determined by F test. After the model equation was established, the optimization program of Design Expert 10 software was used to optimize and analyze several responses. By maximizing the conversion of the expected function, the optimal condition set was finally obtained. According to Wang, et al.^1^, it was necessary to optimize the response surface analysis. The model equations of digestive and respiratory metabolism were optimized according to the method of Montgomery^15^.

## Data Availability

The datasets generated during and analysed during the current study are available from the corresponding author on reasonable request.

## References

[1] Wang H, Zhu X, Wang Y, Luo M, Liu Z (2012). Determination of optimum temperature and salinity for fertilization and hatching in the Chinese pearl oyster *Pinctada martensii* (Dunker). Aquaculture.

[2] Wada, K. T. The Pearl Oyster, *Pinctada Fucata* (Gould)(Family Pteriidae). In *Estuarine marine bivalve mollusk culture*, 245–260 (2018).

[3] Grant J (1996). The relationship of bioenergetics and the environment to the field growth of cultured bivalves. J. Exp. Mar. Biol. Ecol..

[4] Kinne O (1964). The effects of temperature and salinity on marine and brackish water animals: 2. Salinity and temperature-salinity combinations. Oceanogr. Mar. Biol. Annu. Rev..

[5] Gunter G (1961). Some relations of estuarine organisms to salinity. Limnol. Oceanogr..

[6] Newell, R. C. & Branch, G. M. In *Advances in Marine Biology*, Vol. 17 (eds J. H. S. Blaxter, Frederick S. Russell, & Maurice Yonge) 329–396 (Academic Press, 1980).

[7] Hostins B, Braga A, Lopes DLA, Wasielesky W, Poersch LH (2015). Effect of temperature on nursery and compensatory growth of pink shrimp Farfantepenaeus brasiliensis reared in a super-intensive biofloc system. Aquacult. Eng..

[8] Uemoto H (1968). Relationship between oxygen consumption by the pearl oyster and its environmental temperature. Bull. Natl Pearl Res. Lab..

[9] Numaguchi, K. & Tanaka, Y. Effects of salinity on mortality and growth of the spat of the pearl oyster, *Pinctada fucata martensii*. *Bull. Natl. Res. Inst. Aquacul.* 41–44 (1986).

[10] Numaguchi, K. & Tanaka, Y. Effects of temperature on mortality and growth of the spat of the pearl oyster, *Pinctada fucata martensii*. *Bull. Natl. Res. Inst. Aquacul.* (1986).

[11] Numaguchi K (1994). Growth and physiological condition of the Japanese pearl oyster, *Pinctada fucata martensii* (Dunker, 1850) in Ohmura Bay, Japan. J. Shellfish Res..

[12] Numaguchi K (1994). Effect of water temperature on the filtration rate of Japanese pearl oyster, *Pinctada fucata martensii*. Aquacul. Sci..

[13] Muhammad G, Atsumi T, Komaru A (2020). The influence of water temperature, salinity and food availability on nacre deposition rates in shells and pearls of Japanese and hybrid pearl oyster, *Pinctada fucata* (Gould, 1850). Aquaculture.

[14] Li S (2015). Morphology and classification of hemocytes in *Pinctada fucata* and their responses to ocean acidification and warming. Fish Shellfish Immunol..

[15] Montgomery DC (2017). Design and analysis of experiments.

[16] O'Connor WA, Lawler NF (2004). Salinity and temperature tolerance of embryos and juveniles of the pearl oyster, *Pinctada imbricata Röding*. Aquaculture.

[17] Box GE (1954). The exploration and exploitation of response surfaces: Some general considerations and examples. Biometrics.

[18] Davis HC (1964). Combined effects of temperature and salinity on development of eggs and growth of larvae of *M. mercenaria* and *C. virginica*. Fish Bull.

[19] Brenko MH, Calabrese A (1969). The combined effects of salinity and temperature on larvae of the mussel *Mytilus edulis*. Mar. Biol..

[20] Lough R, Gonor J (1973). A response-surface approach to the combined effects of temperature and salinity on the larval development of *Adula californiensis* (Pelecypoda: Mytilidae). II. Long-term larval survival and growth in relation to respiration. Mar. Biol..

[21] Cain T (1973). The combined effects of temperature and salinity on embryos and larvae of the clam *Rangia cuneata*. Mar. Biol..

[22] Lough RG (1975). A reevaluation of the combined effects of temperature and salinity on survival and growth of bivalve larvae using response surface techniques. Fish. Bull.

[23] Tettelbach S, Rhodes E (1981). Combined effects of temperature and salinity on embryos and larvae of the northern bay scallop *Argopecten irradians irradians*. Mar. Biol..

[24] Robert R, His E, Dinet A (1988). Combined effects of temperature and salinity on fed and starved larvae of the European flat oyster *Ostrea edulis*. Mar. Biol..

[25] His E, Robert R, Dinet A (1989). Combined effects of temperature and salinity on fed and starved larvae of the Mediterranean mussel *Mytilus galloprovincialis* and the Japanese oyster *Crassostrea gigas*. Mar. Biol..

[26] Lu W, Li W, Ke C, Wang H (2017). Reproductive success under the joint influences of temperature and salinity in noble scallop, *Chlamys nobilis* (Reeve). Aquac. Res..

[27] Southgate, P. C. *et al.* In *The Pearl Oyster* (eds. Paul C. Southgate & John S. Lucas) 303–355 (Elsevier, 2008).

[28] Yang J (2022). Physical responses of *Pinctada fucata* to salinity stress. Front. Mar. Sci..

[29] Sun J (2021). A comparative study on low and high salinity tolerance of two strains of *Pinctada fucata*. Front. Mar. Sci..

[30] Lannig G, Eilers S, Pörtner HO, Sokolova IM, Bock C (2010). Impact of ocean acidification on energy metabolism of oyster, *Crassostrea gigas*—changes in metabolic pathways and thermal response. Mar. Drugs.

[31] Ivanina AV (2013). Interactive effects of elevated temperature and CO2 levels on energy metabolism and biomineralization of marine bivalves *Crassostrea virginica* and *Mercenaria mercenaria*. Comp. Biochem. Physiol. A: Mol. Integr. Physiol..

[32] Ghiselli A, Serafini M, Natella F, Scaccini C (2000). Total antioxidant capacity as a tool to assess redox status: Critical view and experimental data. Free Radical Biol. Med..

[33] Liu, X. & Yan, A. Recovery and adaptation process of *Pelteobagrus fulvidraco* in the experimental circulatory system after transportation stress. *J. Fish. China*, 495–501(2006), https://kns.cnki.net/kcms/detail/detail.aspx?FileName=SCKX200604010&DbName=CJFQ2006

[34] Ocaño-Higuera VM (2011). Effect of emerged shipment on the physiological condition of the adductor muscle in adult giant lion's paw scallop *Nodipecten subnodosus* (Sowerby, 1835). Aquacul. Res..

[35] Hui W (2021). Growth and survival of Pinctada martensii (Dunker) postlarvae under concurrent variation in temperature, algal ration and stocking density. Aquacul. Rep..

[36] Li S (2016). Interactive effects of seawater acidification and elevated temperature on the transcriptome and biomineralization in the pearl oyster *Pinctada fucata*. Environ. Sci. Technol..

[37] Wang T (2018). Effects of gonadal preoperative treatment on the physiological metabolism of the pearl oyster *Pinctada martensii*: Implications for pearl production. J. Shellfish Res..

[38] Li J (2016). Co-expression of heat shock protein (HSP) 40 and HSP70 in *Pinctada martensii* response to thermal, low salinity and bacterial challenges. Fish Shellfish Immunol..

[39] Wang Y-N (2012). Joint effects of water temperature and salinity on the expression of gill Hsp70 gene in *Pinctada martensii* (Dunker). J. Appl. Ecol..

[40] Wang H (2019). Optimal ammonia concentration for fertilization success in *Pinctada martensii* (Dunker) under the simultaneous influence of temperature and salinity. Aquaculture.

[41] Yang H, Tong S, Wang Z (1998). An review: Digestive enzymes in fishery animals. J. Dali. Ocean Univ..

[42] Lv, H., Liu, J., Chen, J., Shen, H. & Wu, Y. Effects of salinity on filtration, ingestion, and assimilation rates of three filter-feeding bivalves in the Yangtze River estuary. *Mar. Sci.* **40**, 10–17. https://kns.cnki.net/kcms/detail/detail.aspx?FileName=HYKX201608002&DbName=CJFQ2016 (2016).

[43] Zhang Y (2020). Analysis of digestive enzyme activity in *Solen grandis* by response surface methodology. Jiangsu Agricul. Sci..

[44] Qian J, Li Z, Chen S (2015). Synergistic effects of temperature and salinity on activities of amylase and Na+/K+-ATPase in juvenile *Scallop Chlamys nobilis *(Reeve). Fish. Sci..

[45] Pillai BR, Diwan A (2002). Effects of acute salinity stress on oxygen consumption and ammonia excretion rates of the marine shrimp *Metapenaeus monoceros*. J. Crustac. Biol..

[46] Li, X., Li, J. & Qu, Y. Effects of salinity on digestive enzyme activity and diurnal variation ofdigestive enzyme activity of young Yellowfin black porgy *Sparus latus*. *Prog. Fish. Sci.* **27**: 40–45. https://kns.cnki.net/kcms/detail/detail.aspx?FileName=HYSC200601007&DbName=CJFQ2006 (2006).

[47] Chiu YN, Benitez LV (1981). Studies on the carbohydrases in the digestive tract of the milkfish *Chanos chanos*. Mar. Biol..

[48] Jiang, Y., Yan, S. & Yan, Z. Effect of temperature and pH on the activity of digestive enzymes in *Haliotis diversicolor Reeve*. *Mar. Sci. ***36**: 11–18. https://kns.cnki.net/kcms/detail/detail.aspx?FileName=HYKX201202003&DbName=CJFQ2012 (2012).

[49] Zhang S, Zhao Y (1997). The studies on the activities of protease and amylase of *chlamys (Azumapecten) farreri*. J. Dali. Ocean Univ..

[50] Liu M (2015). Effects of Temperature and Salinity on Enzymatic Activity of Digestive and Immunity in Lutraria sieboldii Reeve.

[51] Li J, Wang Q, Du X, Zhang S (2011). Effects of temperature and pH on digestive enzyme activities in hepatopancreas of pearl oyster *Pinctada martensii*. Fish. Sci..

[52] Liu W, Li P (1988). Studies on digestive enzymes of marine Mollusc L. preliminary assay and applications of the digestive enzy mes of m ytilus edulis linne charybdis japonica and littorina sp. Periodical Ocean Univ. China..

[53] Li, X., Li, J. & Qu, Y. Effect of temperature on the activity of major digestive enzymes in yellow fin black porgy (*Sparus latus*). *South China Fish. Sci.* 43–48, (2006) https://kns.cnki.net/kcms/detail/detail.aspx?FileName=NFSC200601011&DbName=CJFQ2006.

[54] Fan D, Pan L, Xiao G, Ma S, Dong S (2003). Effects of temperature and PH on the digestive enzyme activities of *Sinonovacula constricta*. Trans. Oceanol. Limnol..

[55] Zhu X (2012). Combined effects of temperature, salinity and ph on the physiological features of the juveniles of Pinctada martensii (Dunker).

[56] Doroudi M, Southgate PC, Mayer R (1999). The combined effects of temperature and salinity on embryos and larvae of the black-lip pearl oyster, Pinctada margaritifera (L.). Aquacul. Res..

[57] Li, J. *et al.* Effects of Temperature and Salinity on the Respiratory Metabolism of Derbio (*Trachinotus ovatus L*). *J. Guangdong Ocean Univ.* **34**: 30–36, https://kns.cnki.net/kcms/detail/detail.aspx?FileName=SHDX201401005&DbName=CJFQ2014 (2014).

[58] Lin CH, Yeh PL, Lee TH (2021). Time-course changes in the regulation of ions and amino acids in the hard clam *Meretrix lusoria* upon lower salinity challenge. J. Exp. Zool. Part A-Ecol. Integr. Physiol..

[59] Yang S (2018). The salinity tolerance of the invasive golden apple snail (*Pomacea canaliculata*). Molluscan Res..

[60] Nie H, Li W, Li D, Li D, Yan X (2018). Effects of temperature and salinity on activities of respiratory metabolism-related enzymes in cockle clam *Clinocardium californiense*. J. Econ. Animal..

[61] Li X, Dong Z, Xue Y, Shen H, Li J (2009). The lmpact of sharp increase in water temperature and hypoxia on activities of acid phosphatase(ACP) and lysozyme (LSZ) in clam *Cyclina sinensis*. Fish. Sci..

[62] Xie, L., Lin, J., Xiao, R. & Zhang, R. Purification and characterization of alkaline phosphatase from *Pinctada fucata*. *Mar. Sci.* 37–40, https://kns.cnki.net/kcms/detail/detail.aspx?FileName=HYKX200010014&DbName=CJFQ2000 (2000).

[63] Li, Y. & Zhang, H. Progress in antioxidant enzymes study of marine invertebrates. *Mar. Sci. Bull.* **37**: 241–253, https://kns.cnki.net/kcms/detail/detail.aspx?FileName=HUTB201803002&DbName=CJFQ2018 (2018).

[64] Adzigbli L, Yu W, Li J, Yang C, Deng Y (2019). Influence of age on pearl production performance, enzymatic activity, and immune-related gene expression of the pearl oyster *Pinctada fucata martensii*. N. Am. J. Aquac..

[65] Gu, Z., Cao, Y., Yang, H. & Jiao, Y. Effect of janus kinase 3 inhibitor on the development of pearl sac and the expression of lmmune-related genes in pearl oyster *Pinctada fucata martensii*. *J. Guangdong Ocean Univ.* **40**: 1–6, https://kns.cnki.net/kcms/detail/detail.aspx?FileName=SHDX202002001&DbName=CJFQ2020 (2020).

[66] Fu Z (2019). Dietary non-protein energy source regulates antioxidant status and immune response of barramundi (*Lates calcarifer*). Fish shellfish Immunol..

[67] Yang J, Hong J, Fu Z, Ma Z (2022). Effects of dietary curcumin on growth and digestive physiology of *Seriola dumerili*. Front. Mar. Sci..

[68] Ryan TP, Morgan J (2007). Modern experimental design. J. Stat. Theory Pract..

